# Exploring the use of solid fuels for cooking and household air pollution in informal settlements through photovoice: The Fuel to Pot study in Ndirande (Malawi) and Mukuru (Kenya)

**DOI:** 10.1371/journal.pone.0316095

**Published:** 2024-12-26

**Authors:** Isabelle Uny, Tracy Chasima, Line Caes, Lusizi Kambalame, Moses V. M. Chamba, Limbani Kalumbi, Fred Orina, Heather Price, Sian Lucas, Maria Nyikuri, Sean Semple, Hellen Meme

**Affiliations:** 1 Institute for Social Marketing and Health, Faculty of Health Sciences and Sport, University of Stirling, Stirling, Scotland, United Kingdom; 2 Department of Environmental Health, Malawi University of Business and Applied Sciences, Blantyre, Malawi; 3 Department of Psychology, Faculty of Natural Sciences, University of Stirling, Stirling, Scotland, United Kingdom; 4 Communication Department, Malawi University of Business and Applied Sciences, Blantyre, Malawi; 5 Nutrition Department, Malawi University of Business and Applied Sciences, Balntyre, Malawi; 6 Centre for Respiratory Diseases Research, Kenya Medical Research Institute, Nairobi, Kenya; 7 Department of Biological and Environmental Sciences, University of Stirling, Stirling, Scotland, United Kingdom; 8 Department of Social Work University of Stirling, Stirling, Scotland, United Kingdom; Tribhuvan University, NEPAL

## Abstract

**Introduction:**

Worldwide, 2.4 billion people rely on solid fuels such as wood or charcoal for cooking, leading to approximately 3.2 million deaths per year from illnesses attributable to household air pollution. Across Africa, household air pollution generated by solid fuel use accounts for nearly 700,000 deaths each year. Most studies to date have focused either household air pollution exposure, its impacts on particular health outcomes or on the efficacy of mitigation interventions. However, the economic, social, and cultural determinants of household air pollution in Africa are still poorly understood. The purpose of this study was to explore people’s experience of using solid fuels for cooking in two informal settlements, Ndirande in Malawi and Mukuru in Kenya, and the associated harms caused by household air pollution.

**Methods:**

We adopted a community-based participatory method, photovoice, which was conducted with 9 participants in Ndirande and 10 participants in Mukuru. Participants took pictures reflecting their experiences and perceptions of household air pollution harms over a two-week period, and later discussed, sorted and analysed those in a series of meetings. Thematic analysis was used to analyse the data.

**Results:**

With their pictures, participants described fuel stacking and switching behaviours in their communities. They described a mix of charcoal, firewood and other biomass fuels use. They also expressed their awareness and perceptions of the harms caused by smoke when cooking. Participants explained the simple behaviours used by residents to minimize the harms of household air pollution to themselves and within their own household. Other themes explored the roles and responsibilities for procuring fuels in the home, and the stated solutions required to address the issues and manage the transition to cleaner fuels in those informal settlements.

**Conclusion:**

This study highlights not only the need to understand the daily life, priorities and concerns of those who use solid fuels on informal settlements, but also the urgency to place them and their experience at the heart of the solutions that will reduce the health harms of household air pollution.

## Introduction

Worldwide, almost 3.6 billion people- 47% of the world’s population- are still exposed to household air pollution (HAP) and suffer major safety risks linked to the use of solid fuels (wood, charcoal, biomass) for cooking [1]. Currently, this leads to approximately 3.2 million deaths per annum (pa) [1, 2] and an estimated 91.5 million disability-adjusted life years globally [2, 3]. A large majority, 86%, of global human exposure to air pollution occurs in homes in low- and middle-income countries [4]. Household air pollution (HAP) is linked to non-communicable diseases and chronic conditions including asthma, stroke, ischemic heart disease, chronic obstructive pulmonary disease, and lung cancer [2, 5–8]. People with low socio-economic status are the most vulnerable to the effects of HAP, with women, children and the elderly particularly at risk of the effect of HAP [9–12]. It is estimated that by 2025 over 1 billion people in sub-Saharan Africa (SSA) will still be relying on polluting fuels for their cooking and heating needs [13].

Across Africa, the HAP generated by solid fuel use accounts for nearly 700,000 deaths each year (10% of the total mortality), with more deaths amongst women [2, 14]. one of most the affected populations are those who reside in informal settlements- defined as settlements where houses have been- temporarily or permanently- built on land without formal planning approval. In Sub-Saharan Africa, 51% of urban populations reside in such informal settlements -also called ‘slums’, a term accepted by residents themselves in Africa [15] as it is in our setting and one we use in this paper interchangeably. In informal settlements, housing is usually more affordable, but water, sanitation, health access and road infrastructures are often inadequate [16]. Informal settlement residents are exposed to numerous health risks from disasters (climate change, fires, floods), disease epidemics, environmental pollution, poor sanitation infrastructure, and violence [17]. Slum dwellers often rely partially or totally on solid fuels for cooking and heating, because cleaner fuels—such as liquified petroleum gas (LPG) or electricity are either unavailable or unaffordable [18–20]. The use of solid fuel and the impact of HAP in informal settlements populations health in SSA is poorly understood, and scarcely researched [18, 21–24].

Most studies on the use and impact of solid fuels on health have either tended to focus on measuring HAP concentration and exposure at household level, or on assessing the impacts of HAP on certain health conditions and health outcomes. Some have attempted to review the effectiveness of such HAP interventions ranging from improved cookstoves (ICS) to behavioral change and education interventions [25–28]. However the effectiveness of those interventions remains unclear and poorly reported, and their impact on health remains fairly limited [26, 29–31]. Moreover, the economic, social, and cultural determinants of HAP in particular contexts are often poorly understood or acknowledged [26, 32]. Understanding those key factors, and their interrelation in determining fuel choice is key to developing contextual, effective and sustainable HAP mitigation interventions. There is limited evidence on the effectiveness of HAP mitigation interventions which may be affordable, effective and culturally acceptable, particularly in informal settlement contexts [20, 33]. Our previous scoping review found limited research on contextualised perceptions of HAP-related health harms [26], and most studies reporting on HAP interventions tend to exclude participation from those who live in informal settlements, save for a notable few [19–21]. Our study specifically aimed to address those multiple gaps.

To understand the complexity of those issues through the lens of those who live in informal settlements, we selected a qualitative participatory methodology called photovoice. This is a visual participatory method originally developed by Wang and Burris, which aimed to enable community participants to document their own issues and assets, in order to support collective advocacy [34, 35]. The method consists broadly in developing or sharing a study goal with participants, recruiting and training participants in the use of cameras so they may take photos themselves which represent the issues of concern to them in their communities. The participants and researchers later come together to analyze the photos and their meaning, to discuss the issues raised, with the end purpose of developing actions or advocacy campaigns which are contextually relevant. We selected this method because it is well established [36–40], and was the most appropriate to understand the issues of solid fuel use for cooking and HAP exposure from the lived experience of informal settlement residents in Ndirande and Mukuru. The photovoice method allows for a unique perspective on solid fuel use and HAP harms at household and local community level [30, 41], and one that is currently too rare in the literature, particularly in informal settlements contexts. The aim of the study was to use participatory and interdisciplinary methods to explore the lived experiences of informal settlement people who cook food with solid fuels in Kenya and Malawi, and the perceived impact on their health. By choosing the photovoice method, we sought to also address a methodological gap in informal settlement population health research. Below, before we present the results, we describe the specific context of the two informal settlements in which the study took place.

### Research context

Our study took place in 2 informal settlements: Mukuru in Kenya, and Ndirande in Malawi. The specific context of each area is briefly presented below.

#### The Mukuru slum (Kenya

The Mukuru slum is located within the urban area of Nairobi in Kenya. Approximately 60% of the population of Nairobi resides in informal settlements, which have expanded significantly since the 1950s. Mukuru consists of approximately 30 villages and the overall population is estimated to range from 300,000 to over 700,000 people. Mukuru Kwa Njenga, the specific area where the study took place has a population estimated at between 100,000 and 150,000 [21, 42, 43]. It is notoriously difficult to estimate slums population as people living there are often unrecorded and transient. Most people living in this area are on low income and unemployment is high. Housing is predominantly informal and often lacks basic amenities. Many people live in small houses made of corrugated iron sheets, mud, or other low-cost materials. These dwellings often have poor sanitation, lack running water and waste disposal. The area faces severe flooding in the rainy season and heavy pollution due to its location near an industrial area and surrounding busy roads. Many of the slum dwellers in Mukuru work as casual labourers in the manufacturing industries close to the slum. Others operate small-scale businesses selling pre-cooked foods and other household items. Common diseases in the informal settlement include diarrhoeal diseases, pneumonia, malaria, tuberculosis and HIV- AIDS; access to public health facilities remains difficult for most [21, 42, 43].

#### The Ndirande Township (Malawi)

The Ndirande Township is one of the largest informal settlements in Malawi, adjacent to Blantyre, a large city in the southern part of Malawi. Ndirande is composed of 3 different wards-: Matope, Makata and Gamulani. Together they hold a population officially recorded at 97,839 [44]. The study took place in the Makata ward. Most parts of the township are overcrowded and have no formal road networks or waste disposal systems. Houses are usually made of bricks, wooden panels and corrugated iron sheets. Unemployment is high and many people are on low income. Ndirande is served by one health centre and is close to an industrial area. The main economic activities for the populations in the area revolve around small jobs in local industries, garages and carpentry, in bottle stores and local markets. In Ndirande–as in Blantyre-access to electricity remains very low [45–47]. Therefore, the main fuel sources for cooking are solid fuels, including charcoal and firewood with people also ‘stacking’ other fuels for their daily needs. This has led to the depletion of forest reserves around the township, exacerbated by overpopulation. Common diseases also affect populations in this informal settlement including diarrhoeal diseases -especially in young children [48]. The added impact of HAP from biomass fuel use in populations around Blantyre have been reported [49–51], as have been common and severe injuries related to cooking- especially burns and scalds-[52]. However, to our knowledge, this study was the first of its kind to take place in the Ndirande informal settlement.

## Methods

Ethical approval was obtained for this study at the University of Stirling (No.1828), at the Malawi University for Business and Applied Sciences (MUBAS) through the National Committee on Research in the Social Sciences and Humanities in Malawi (No. P.11/21/607) and from the Kenya Medical Research Institute (KEMRI/SERU/CRDR/067/4350). The Malawi research team who conducted the research in Ndirande was fluent in Chichewa–the local language in this part of the country-as well as English. The Kenya Team -who conducted the research in Mukuru- spoke Swahili as well as English.

Prior to recruitment, the researchers conducted the necessary entry and sensitization meetings with the local Traditional Chiefs and authorities in the community, to introduce the topic of the study and the proposed methods. A checklist with inclusion and exclusion criteria was devised by the Team. A community meeting was called at which researchers explained to residents the objectives of the project and encouraged residents to come forward. Inclusion criteria were broad so as to allow for inclusivity, and the only exclusion criteria were being under 16 years of age, fully visually impaired or lacking mental capacity to fully understand the information and consent form and to undertake the photovoice. Interested participants who met inclusion criteria were given the information sheet and consent form and sufficient time to ask questions to the researchers. Within this small sample–which is advocated for photovoice studies [34, 35] -we aimed to have a purposive representation of male and female participants and younger and older participants as is typical in the population in Ndirande and Mukuru as a whole, and as has been done elsewhere [30]. All of those who agreed to take part completed a consent form; young people [16–18] filled an assent form and their parents a consent form to enable them to take part. No young people were recruited in Mukuru, as the parents of the young people who came forward did not wish to give consent because their feared the photovoice activities could distract their children from their schooling and other household duties. The participants characteristics are described in Table 1 below.

**Table 1:** Participants characteristics.

Participants characteristics	Ndirande	Mukuru
**Age**		
** 16–18**	3	0
** over 18**	6	10
**Gender**		
** Female**	**6** (Participant ID number*] M01; M04; M05; M06; M09; M10)	**6** (Participant ID number* K02; K06; K07; K08: K09; K10)
** Male**	**3** (Participant ID number*: M03; M07; M08)	**4** (Participant ID number*: K01; K03;K04; K05)
**Total**	**9**	**10**

* as labelled in the paper and figures

All personal data regarding the participants was kept on the partner institutions secure server, as per the partner institutions’ data protection policies. A two-stage consent process was used for those who participated in the photovoice -those who took the pictures- at the start of the study (written consent) and towards the end of the study (verbally, relating to disclosure and sharing of photographic material). During the initial training, participants [those taking pictures in their community] were trained on how to respect privacy through their picture taking, and not to take pictures of people or their houses if the person expressed their refusal. All the people pictured by the photovoice participants (family members or neighbours/ community members) asked for their photo to be taken and gave written consent in the form of a third party consent. The study information was given to all of them in the form of a large, printed postcard with key info on the study, and their written consent recorded on it [one postcard was given to the person asking for their picture to be taken; the other -with their signature- gathered by the photovoice participant taking the picture and passed on to the Researchers [MN,TC]. The template of the third party consent postcard [in English; prior to translation for use in Ndirande and Mukuru] has been added as a S1 File. Those consent postcards were securely stored at MUBAS and KEMRI, by the researchers.

Photovoice participants also reiterated- through collective verbal consent- in Feb 2023 at the end of the study during community dissemination events in Mukuru and Ndirande- their consent for the pictures that they had selected from the photovoice to be used in journal articles and other dissemination outputs. Therefore, all individuals in this manuscript have given written informed consent (as outlined in the PLOS consent form guidelines) to publish the images used in the manuscript.

### Data collection

**Fig 1:**
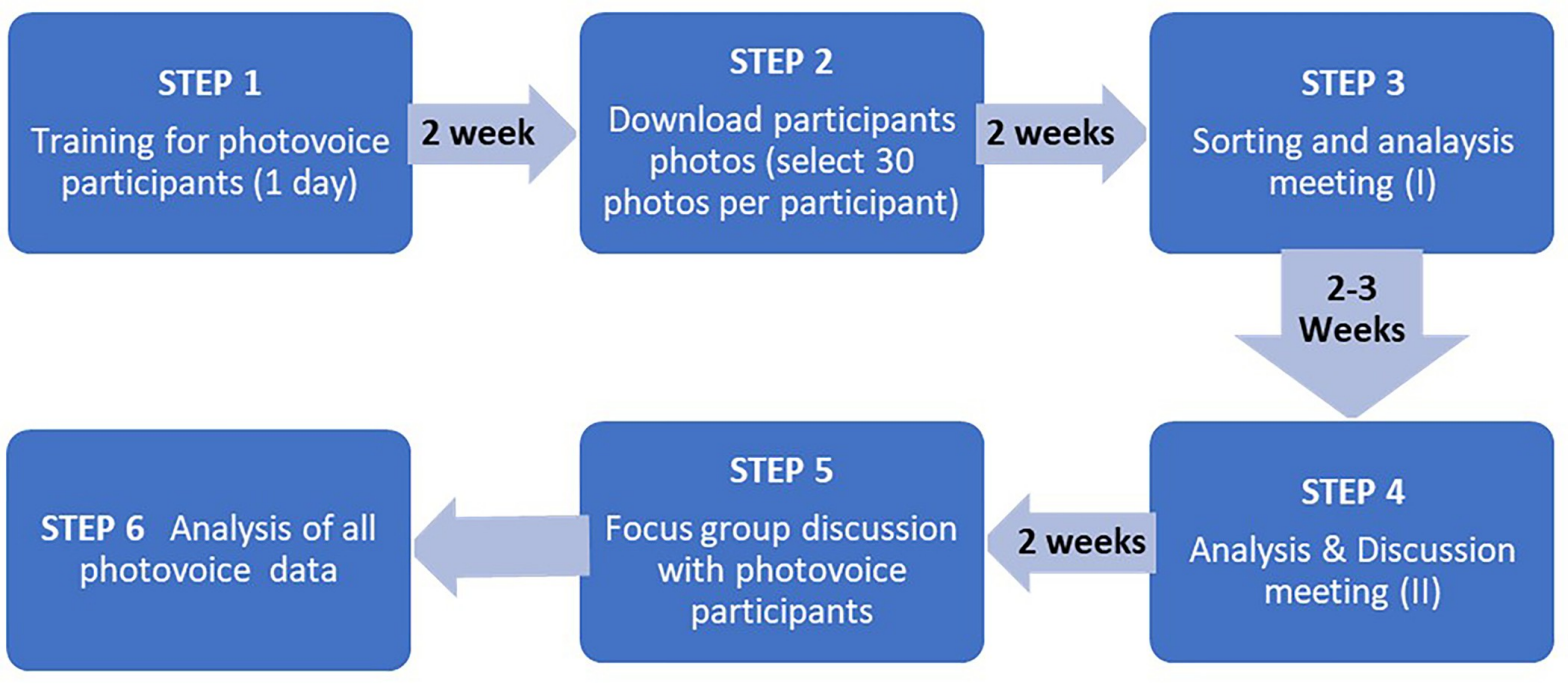
Steps of the photovoice method.

In the last thirty years, photovoice has been undertaken in many different ways including online more recently [37, 38, 53]. In Fig 1 we describe in detail the steps we used in this photovoice study and thereafter we elaborate on each step used in our own process. This photovoice study took place in both countries between June and September 2022.

At the very start and before fieldwork, the entire Fuel to Pot research team was trained by an expert in photovoice methodology who had led a recent photovoice study in Malawi around HAP [29, 30, 54]. This consultant and the lead author (IU) developed a bespoke photovoice training manual for the research Teams in each country.

Each country team also developed its own participant’s manual for participants, covering the study topic, straightforward and pictorial instructions on picture taking and issues of privacy and confidentiality when taking pictures in one’s neighbourhood and community. This manual was used as part of the participants’ training (Step 1). The participants were equipped with basic android phones to take photos and equipped with data and funds to charge the phones for the duration of the study. Participants were compensated for travel cost and time at each step of the photovoice methodology (and were gifted the phones as a thank you gesture at the end of the study). After their initial training, the participants were given a period of 2 weeks to take photos and self-select a maximum of 30 pictures related to the issue of solid fuel use and cooking in their neighbourhood areas. At Step 2, the participants met with the research assistant (RA) and photos were downloaded onto a password-protected laptop before being printed and re-distributed to the participants for the first sorting and analysis meeting (Step 3). In Ndirande the discussions at Steps 3 and 4 were recorded and transcribed. In Mukuru, participants did not wish for discussions to be recorded, and therefore notes were taken by the researchers to support the analysis. At the first sorting meeting each participant was given a printout of all of their photos. Each presented those and discussed them with the rest of their group. Participants started to sort all the photos into collectively agreed broad themes at Step 3. At Step 4, the second analysis and discussion meeting, the participants reviewed their agreed themes and photo groupings. They discussed the problems and advantages of each solid fuel, as well as potential solutions to mitigate HAP harms in their community. To guide and facilitate the discussions in meetings at Steps 3 and 4, we adapted the SHOWED model below developed by Ardrey *et al* [29] presented in Table 2.

**Table 2:** The SHOWED model (adapted from Ardrey *et al* [29]).

**S**	**What do you See here?**
Explain what the eye sees; describe the images.	
**H**	**What’s really Happening here?**
Talk about the unseen story of the images, think about how you would explain this to someone from outside your community. If there are people in the images, what is their role in the cooking process?	
**O**	**How does this relate to Our lives?**
Explain what these images say about life in your household, in your village, in the wider community. Why did you take this particular image? Explain what you want others to understand when viewing the image.	
**W**	**Why does this problem, concern, or strength exist?**
Talk about why things happen in this way. Has this always happened or have changes occurred over time? Does the image show usual or unusual behaviour?	
**E**	**Can you Explain more about the role of the people?**
Explain more about the role of people in the images; who is doing what and why is that particular person involved in the activity?	
**D**	**What can we Do about it?**
If these images can be used to illustrate something positive about cooking on solid fuel, explain how. If the images show something that could be improved, then suggest how this could be done. Explore who has the power to facilitate any change in cooking practices/ fuel use	

The participants themselves selected and agreed which photos to use in this paper (more pictures were selected than was possible to include below in findings). In Step 5, we conducted a focus group with the participants to explore their experience of taking part in a photovoice study, which will be reported on in another paper.

### Final thematic analysis

In Step 6, the notes and transcripts from the analysis meetings with participants were transcribed and translated. The researchers in each country placed all the photos selected by participants as well as their comments into a collated PowerPoint document by themes. We used a thematic analysis to analyse the data, as described by Braun and Clarke [55–57]. All transcripts and PowerPoint documents were de-identified and imported into the NVivo 20 software to support analysis. Two researchers from the Team [IU, LC] reviewed the documents and developed a thematic coding framework. The coding was undertaken by three members of the research team [IU, LC, TC]. Analysis started by close reading of the transcripts and notes alongside close review of the photos selected by participants. We used a framework matrix approach in Nvivo to thematically summarise the data. The whole research team discussed those themes and sub-themes at several meetings and related those to the photos selected, narrowing down the themes presented in the results. From those discussions and anlysis, LC and IU wrote more analytical thematic memos, which formed the basis of the first draft of the paper, reviewed thereafter several times by the whole team.

## Results

In this section we discuss findings and present photos and quotations from the analysis meetings with participants (Step 3 and 4 of the photovoice process described in Fig 1). For each theme the photo tiles show Mukuru-(Kenya) pictures at the top and Ndirande (Malawi) at the bottom, with the number and provenance of participants clearly labelled: e.g. Kenya participant 01 is labelled K01, Malawi participant 01 is labelled M01. Quotes corresponding to the pictures from participants are included where possible. Other quotes- labelled similarly- from other participants are used throughout the paper.

### Fuel stacking in slum environments

**Fig 2:**
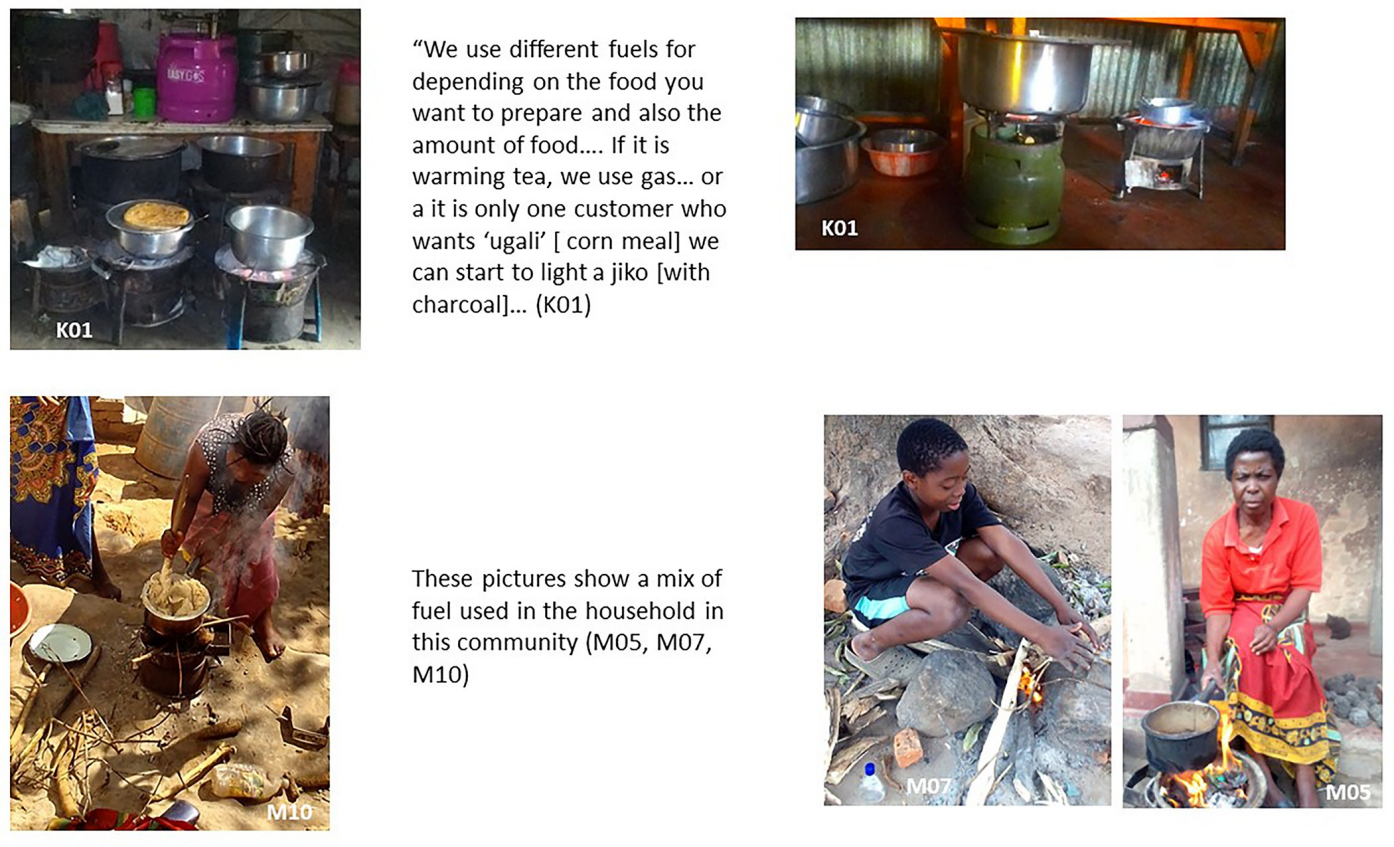
Fuel stacking.

This study focuses on the use of solid fuels (such as charcoal, wood and other biomass fuels) because of the resulting damaging impact of HAP on health. Participants described the type of stove and fuel stacking practiced by residents in the informal settlements of Mukuru and Ndirande (Fig 2). The term ‘fuel stacking’ [58] refers to the concept of using multiple fuels (and stoves) or a combination of fuels at the same time to meet households’ needs for cooking. Fuel and stove stacking- or switching- are often dictated by fuel costs, fuel availability, personal needs and preference, cultural norms, and time availability to cook [59–62]. In the two informal settlements of Mukuru and Ndirande, the most commonly used solid fuels stacked were wood, charcoal and charcoal briquettes (also called charcoal balls locally). However, several other non-solid fuels were also used at the same time (depending on seasonality, availability, price).

In Mukuru, these other non-solid fuels were paraffin, kerosene and liquified petroleum gas (LPG). Participants mentioned that some people in the slum were also able to use electricity for cooking. The use of non-solid fuel was much more common in Mukuru. Whereas in Ndirande non-solid fuel use was barely mentioned by participants and the use of electricity was rare. In both research sites however, electricity supply remains unreliable, and electricity was mainly used by more affluent households who could afford to purchase both the energy and the appliances. In Mukuru, kerosene was also used due to its low cost (20 KS-0.15$- per litre), and its availability for purchase at petrol filling stations. LPG gas was also used commonly in Mukuru and preferred by participants. However, its cost was considered to be prohibitive (participants reported that a 6 Kg gas cannister ranged in price from 1300 to 1700 KS- circa $10). However recently in Mukuru, LPG companies have been able to instal metered cylinders in some residents’ houses, where the gas supply can be topped up by paying using mobile phone credit or pay-as-you go options [63].

When discussing these issues of fuel stacking in a meeting, a participant in Ndirande summarised the situation as follows:

*“This depends on one’s financial status. Some can manage to cook using electricity if they have money to buy the electricity and electric appliances, while others may not manage to buy electricity and electric appliances and may find only charcoal affordable. " (M01;Female)*

Below we discuss in turn, the array of solid fuels used in Mukuru and Ndirande, and their advantages and drawbacks, as perceived by the photovoice participants themselves, through their own pictures and words.

### Firewood use: Advantages and drawbacks

**Fig 3:**
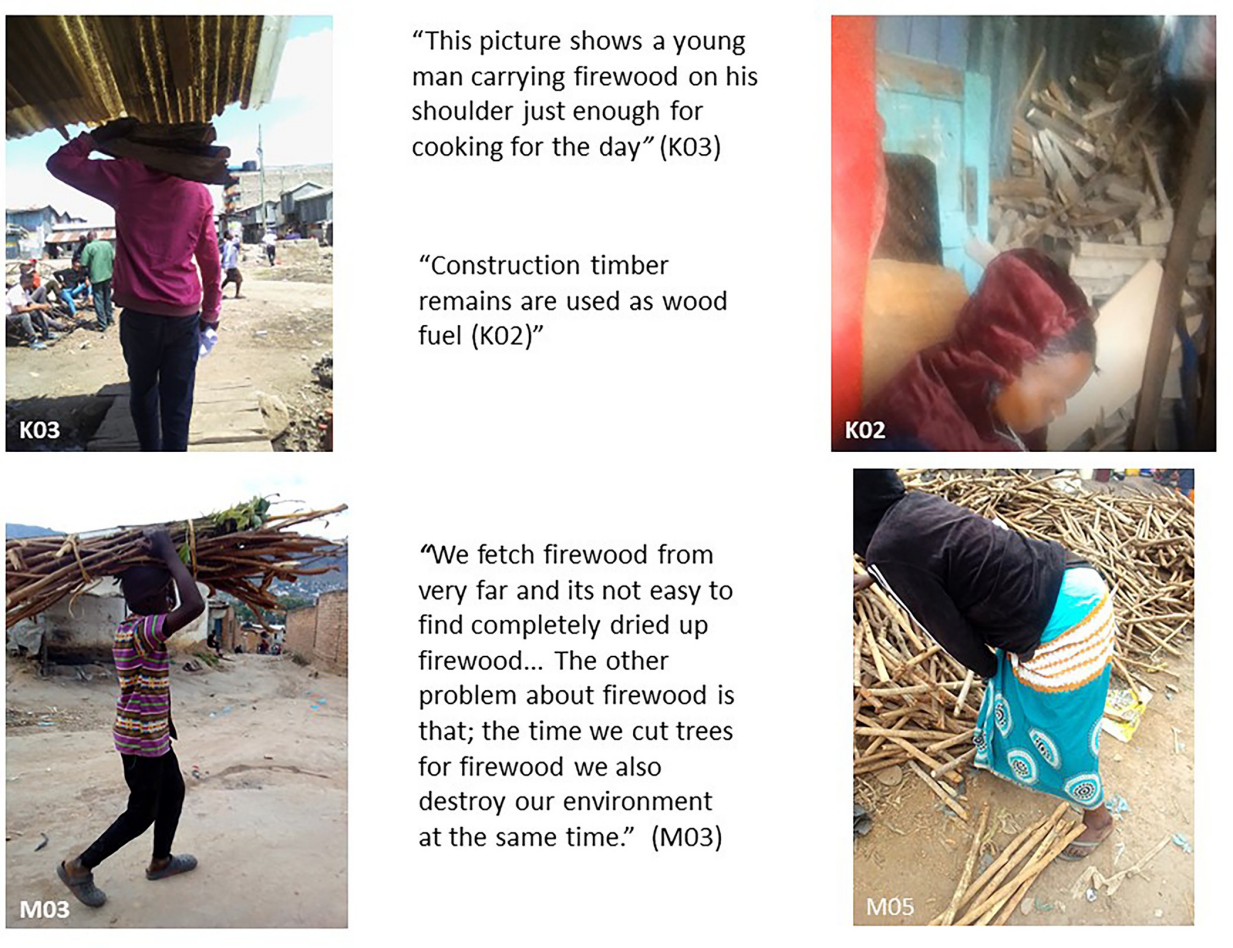
Firewood usage.

Our photovoice findings showed that firewood is used in both informal settlements, albeit procured or collected in different ways (Fig 3).

**Fig 4:**
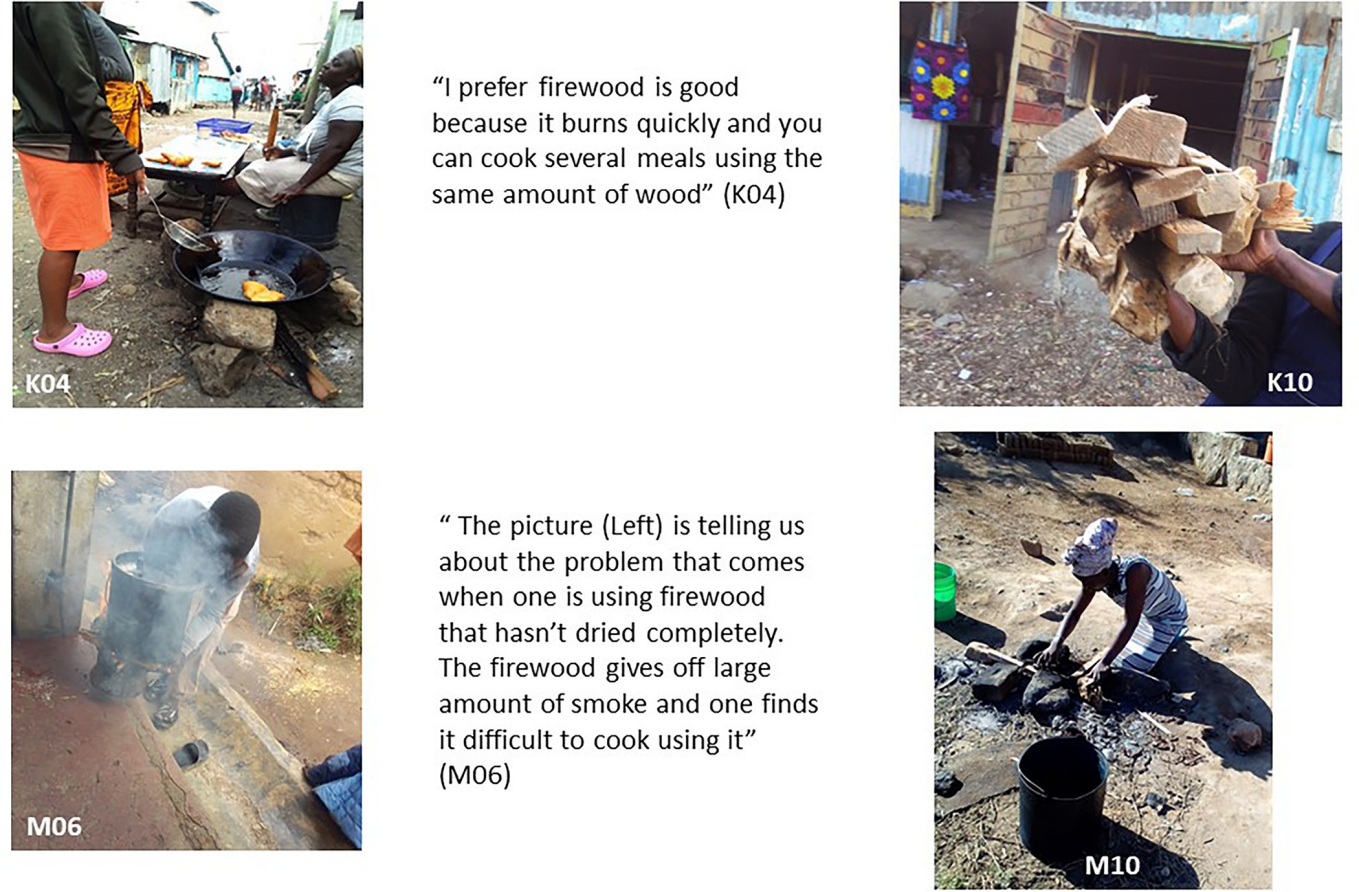
Firewood advantages and disadvantages.

In Mukuru firewood is mostly procured from construction timber residue and off-cuts which are used as fuel and bought at the timber yard or collected at the carpentry workshops. In Ndirande, firewood can still be collected in some places in the wild, but people at times need to walk long distances to forested areas in the surrounding hills to collect firewood to use as fuel; Residents sometimes pay to collect residual firewood on other people’s land. However, in Ndirande participants stressed that collecting wood is becoming a challenge due to deforestation. A number of advantages and disadvantages with this fuel source was alsomentioned by participants (Fig 4).

As shown in the figure above, in both locations, firewood is often burnt on three stone fireplaces or charcoal burners (called ‘jiko’ in Kenya; and ‘mbaula’ in Malawi). Firewood is seen as a useful cooking fuel because it is quick to light, burns intensely and enables fast cooking. Some food vendors in Mukuru used wood for instance to cook fast foods and snacks sold for breakfast to residents passing by in the early morning rush (K04, in Fig 4). Wood was also perceived to make the food taste good by participants. Participants described the main advantages of firewood as being its low cost compared to other fuels (especially in Ndirande where residents can still collect some of it for free at times).

However, participants in the study highlighted that the key disadvantage of firewood is that it produces a lot of smoke (particularly when the wood is damp). This is damaging to health when cooking indoors or near to the house (e.g. on the veranda); firewood also produces soot which also makes the house dirty and unsanitary. Therefore, participants explained that, as far as feasible, wood has to be burnt outside or in the open preferably when cooking. In Ndirande, this was described as a challenge during the rainy season, when people find it hard to cook outdoors, whilst in Mukuru the key challenge to using firewood outdoors was the lack of spaces between houses (this may explain why it is used more by vendors in the open in Mukuru where there is more space at the stalls rather then in individual houses).

### Charcoal and charcoal briquettes usage

**Fig 5:**
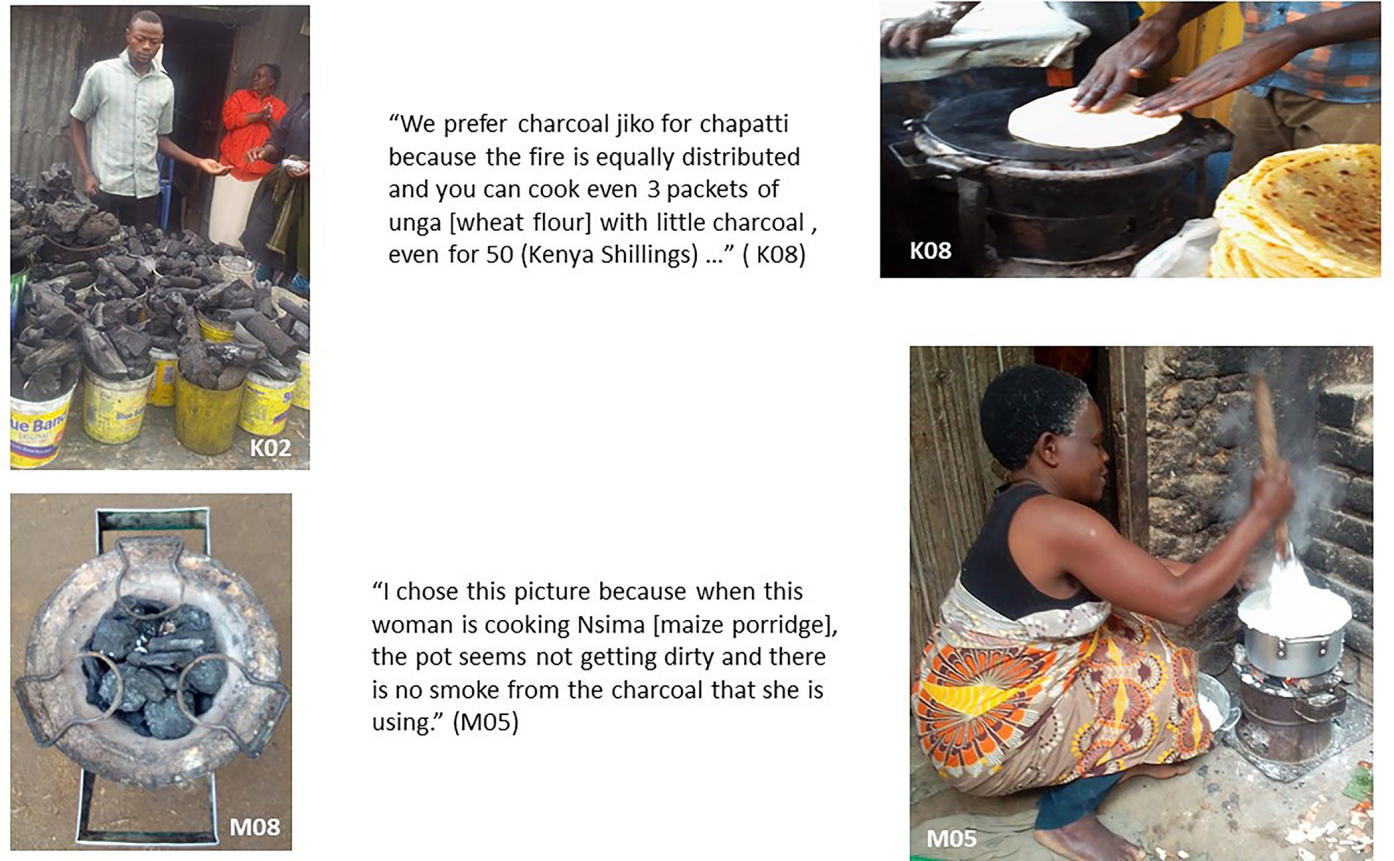
Charcoal procurement and usage.

In both countries, charcoal was available and commonly used. Participants explained that It was usually bought from charcoal vendors either in larger bags or in small tins, according to household needs and available income (K02, see Fig 5 below). Participants in Mukuru and Ndirande expressed that a main advantage of charcoal is that a small amount can be used to prepare several meals; charcoal burns more slowly and can keep food warm for longer. Moreover, charcoal, in both settings, is readily available. Participants in Malawi explained that charcoal could be used for heating indoors- in the cool season-, because it does not produce as much smoke; and the fire requires less constant ‘feeding’.

**Fig 6:**
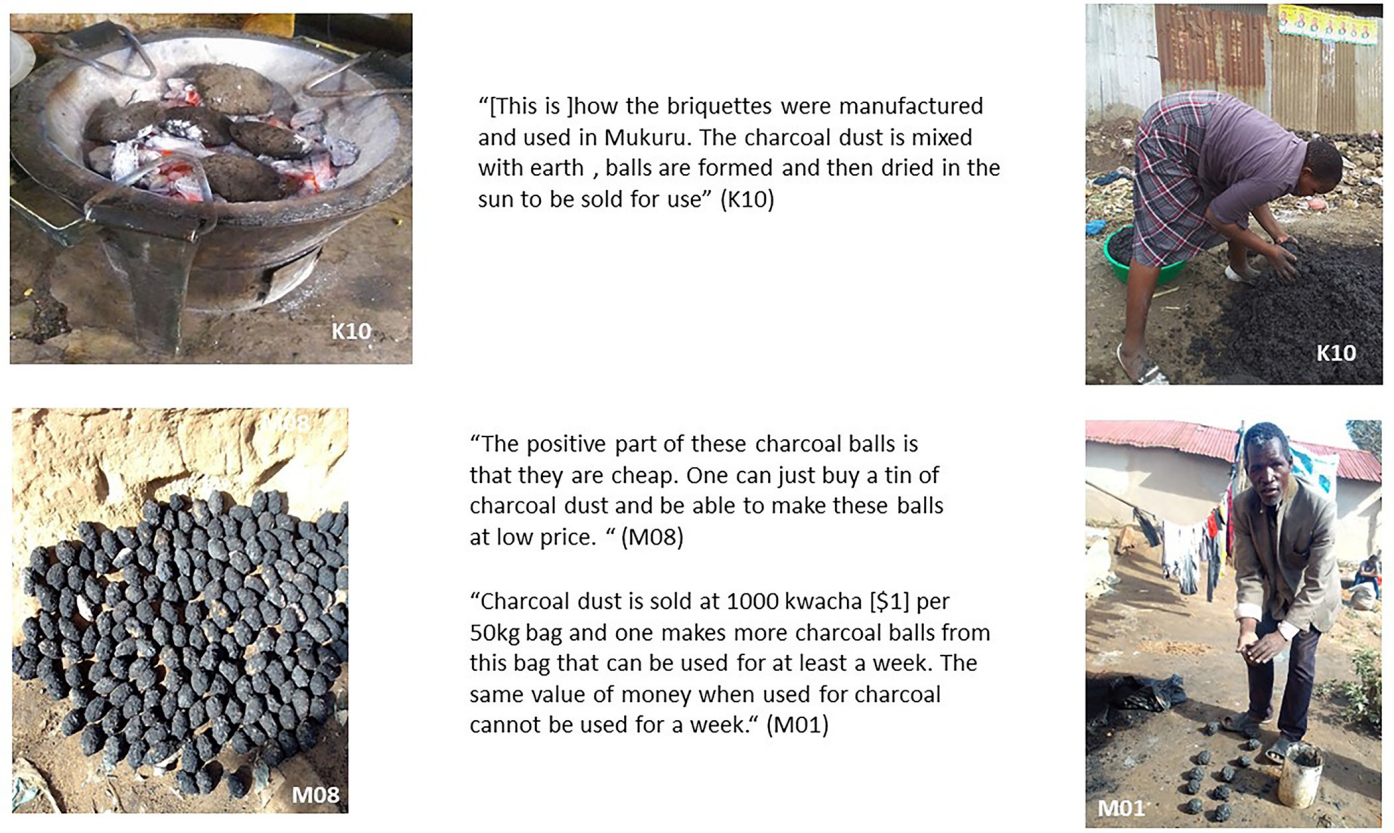
Charcoal briquettes.

A more recent phenomenon which our study findings highlighted in both countries is the use of charcoal briquettes (also known as charcoal balls, pictured in Fig 6). These are made with charcoal dust either bought or gathered from charcoal vendors (in larger bags), which is then mixed with earth or sand, and left to dry in the sun before being used as fuel. These briquettes are now used widely in both locations; they are usually added to charcoal to make the fuel last longer and thus help households to save on fuel costs because these briquettes are cheap. In Ndirande, charcoal balls were sometimes used to cook indoors once the fire is lit and the smoke has subsided to help keep the house warm. However, in both Ndirande and Mukuru, participants expressed that charcoal balls also require the use of other ‘starter’ fuels to be lit when used on their own, which in turn may release more smoke. Some residents used discarded plastic bottles to start the charcoal burning process. Ndirande participants explained that charcoal briquettes, often made by the household members themselves, can be hard to make and store during the rainy season, as they may melt and require drying for long periods in the sun to be useable; dry space or dry storage was often hard to find for this activity in the rainy season.

Although charcoal is still available in both countries, participants acknowledged that there is a supply shortage. In Ndirande, there is high demand for charcoal during the rainy season. While available and used in both settlements, charcoal briquettes seemed to be more commonly used in Ndirande, where residents increasingly need to economise on their fuel costs.

### Other solid fuels as a last resort

**Fig 7:**
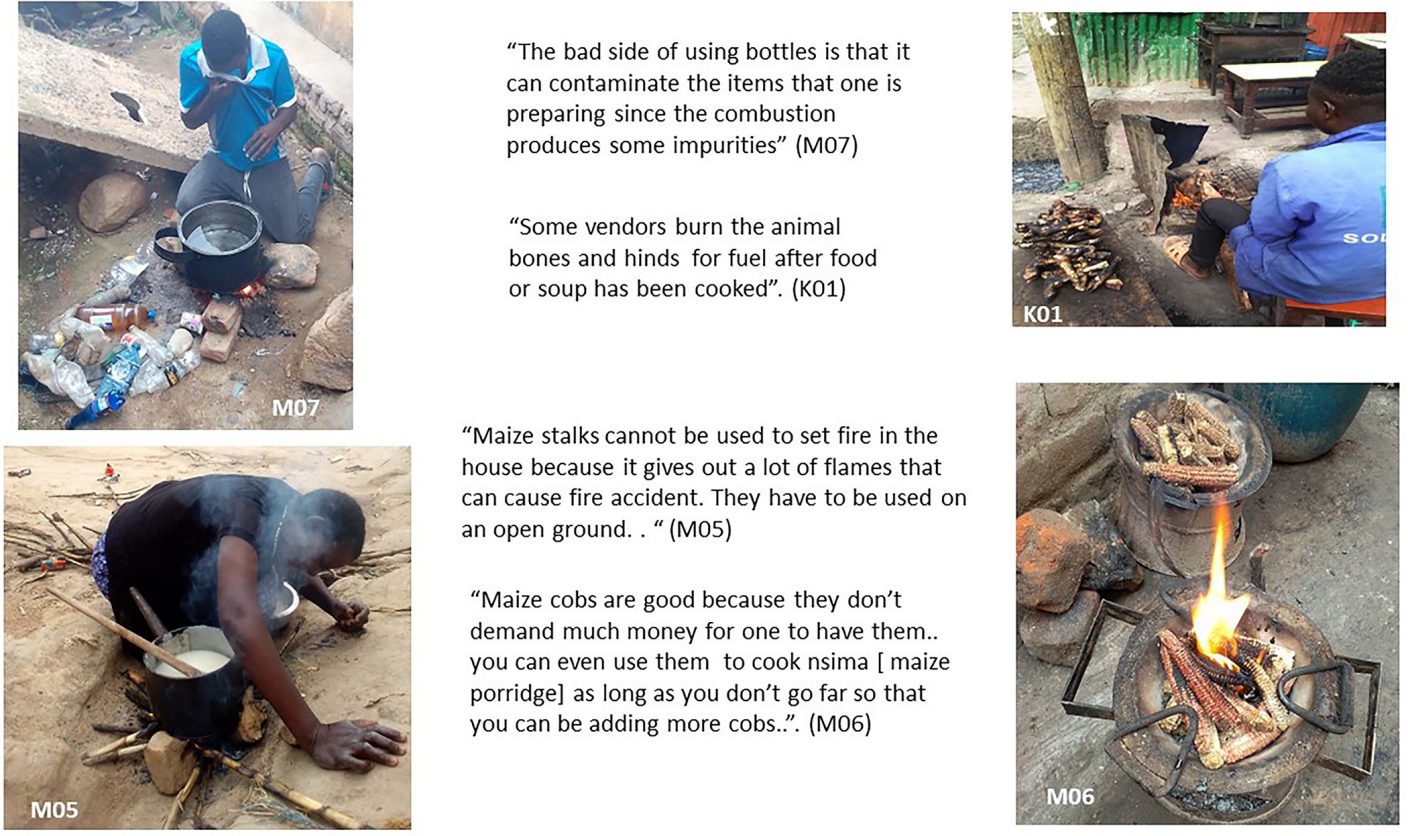
‘Fuels of the last resort’.

What we named ‘fuels of the last resort’ are the solid fuels residents use when they have no other fuel available or when families cannot afford to purchase any other fuels. Some are shown in Fig 7. These fuel sources are often opportunistic, based on what is available at the time, in the season, or in the vicinity of the slum, as is keenly explained by Ndirande participants below:

“*Maize stalks is the last resolution that one takes when other means used for cooking such as firewood, charcoal and charcoal balls for instance are out of reach due to financial problems and other related reasons*” (M03, male)

“*Because of our financial status*, *we may not manage to buy charcoal and firewood all the time that’s why we use these maize stalks at some point in time”* (M08; male)

In Mukuru, some waste products were occasionally as added to other fuels by vendors after those had been used in cooking food (e.g.bones and hinds, after cow or goat’s meat has been used to make soup for instance). In Ndirande the main fuel of last resort was often maize residues (cobs or stalks). Maize stalks and cobs were perceived to give a less noxious smell or smoke than plastic when cooking. Maize stalks were seen to burn fast and give off intense heat and therefore were seen as useful for fast cooking (or heating water for bathing). These maize stalks and cobs can also be gathered from fields after the harvest season for free around Ndirande, which is surrounded by agricultural land, unlike Mukuru. However, as maize stalks are only available during the harvest season, the use of plastic waste as fuel of the last resort, especially plastic bottles which are available year-round and collected on the street, was also common in Ndirande. Plastic bottles, plastic wrapping paper or plastic bags can be gathered in the slum in the streets; disused plastic bottles can also be bought from people who gather them in the community. Such plastic items can be either used as a fuel on their own, or in combination with other fuel sources, such as quick starter to start a charcoal fire. However participants in Ndirande stressed that using plastic bottles for fuel is very unhealthy as the smoke they produce is thick, and noxious (M07, Fig 7).

### Smoke and household air pollution harms from cooking on solid fuels

#### Perception of the health harms of smoke inhalation

**Fig 8:**
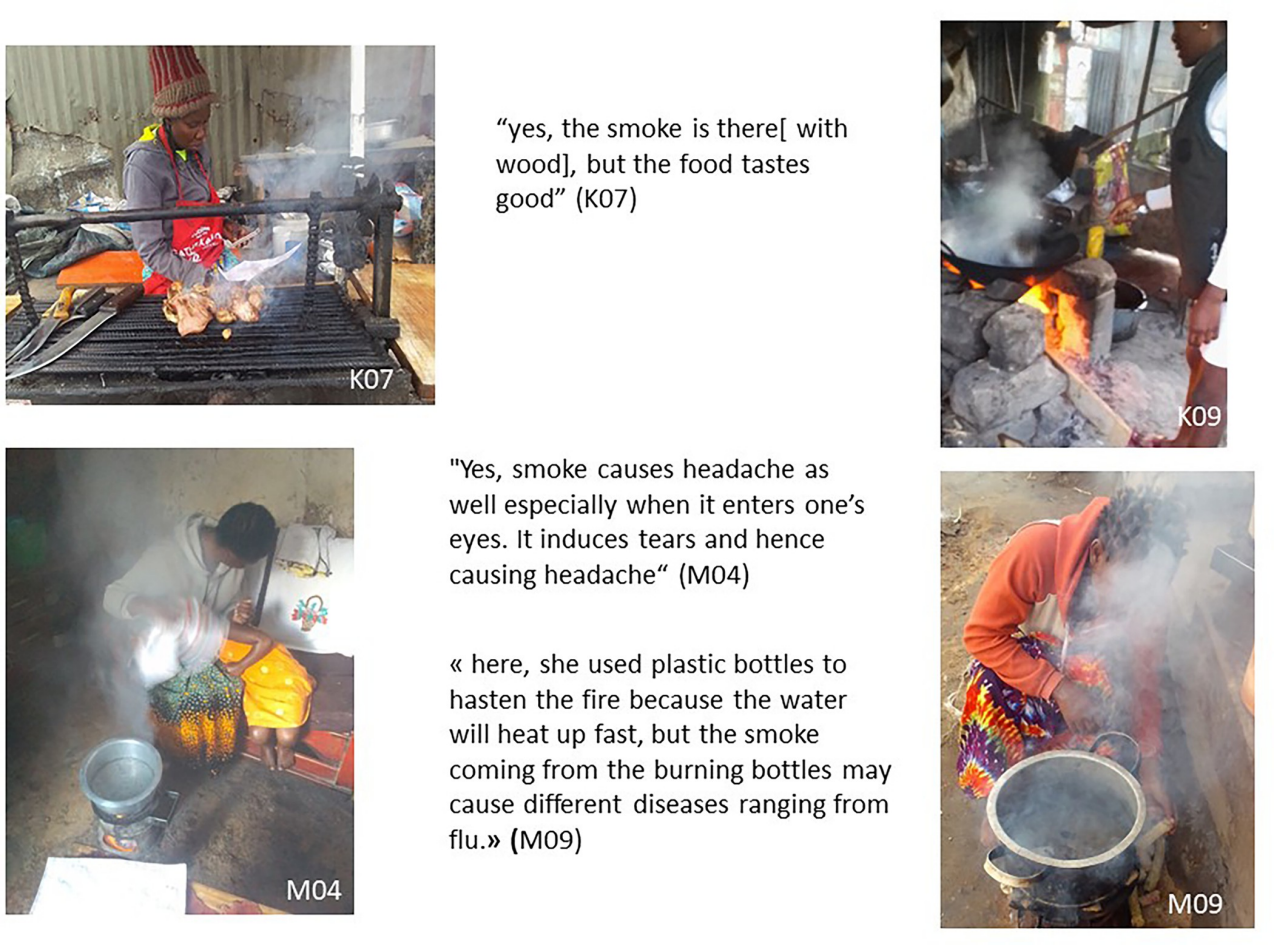
The smoke and health harms.

Smoke and the dangers of smoke inhalation to health were discussed by photovoice participants in each informal settlement (Fig 8). Most of them reported that smoke causes headaches, coughing, impacts on the eyes and with repeated exposure can even lead to heart attacks, as expressed below.

“*Firewood becomes bad when one is exposed to it for long time whereby the smoke from the firewood enters her heart” (M06, female)*

“*Smoke can also hurt our bodies*. *People are sick from different ailments some which have been caused by smoke*. *Some of these ailments include lung diseases that is also caused by smoke” (M08*, *male)*

Participants were particularly concerned with the impact of smoke exposure on women—who are often the main cooks- and children, who may have a role in lighting fires and cooking or who may simply at times be playing nearby stoves when cooking is taking place. Participants perceived that some solid fuels (damp wood, plastic bottles, cartons, maize residues) create more damaging smoke which is bad for their health.

*“All these issues come in due to smoke that is released by firewood that has not completely dried up" (M08, male)*

*“the smoke coming from the burning bottles may cause different diseases ranging from flu”(M09*, *female)*

**Fig 9:**
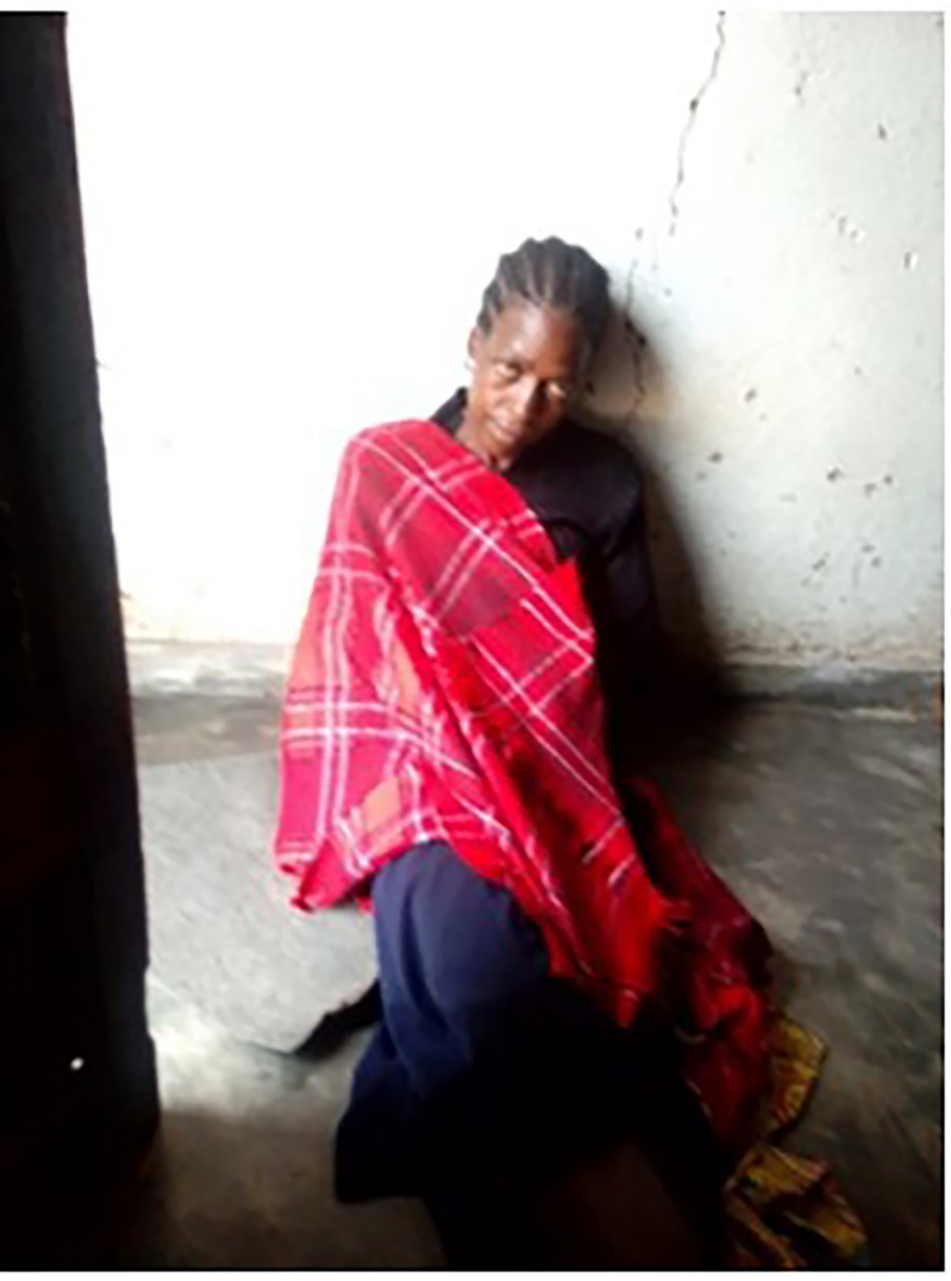
CO exposure and dangers.

Some study participants also mentioned the dangers of the more invisible carbon monoxide (CO) emanating from the burning of charcoal in burners inside confined houses (Fig 9). Although this danger was not commonly recognised within communities. In Ndirande, a participant explained that fainting can occur when charcoal is used indoors without proper ventilation, and told the following story to illustrate the photo appended below:

“*The woman (shown in the photo below) was asleep when a child set a charcoal burner alight and later went to school leaving behind smouldering charcoal. When people came to this house found the woman in a comma (fainted) after breathing charcoal smoke. The charcoal burner was removed, and the woman was assisted. From this, we can see that the smoke from smouldering charcoal is dangerous as it caused the woman to faint upon breathing the smoke.” (M06; female)*

**Fig 10:**
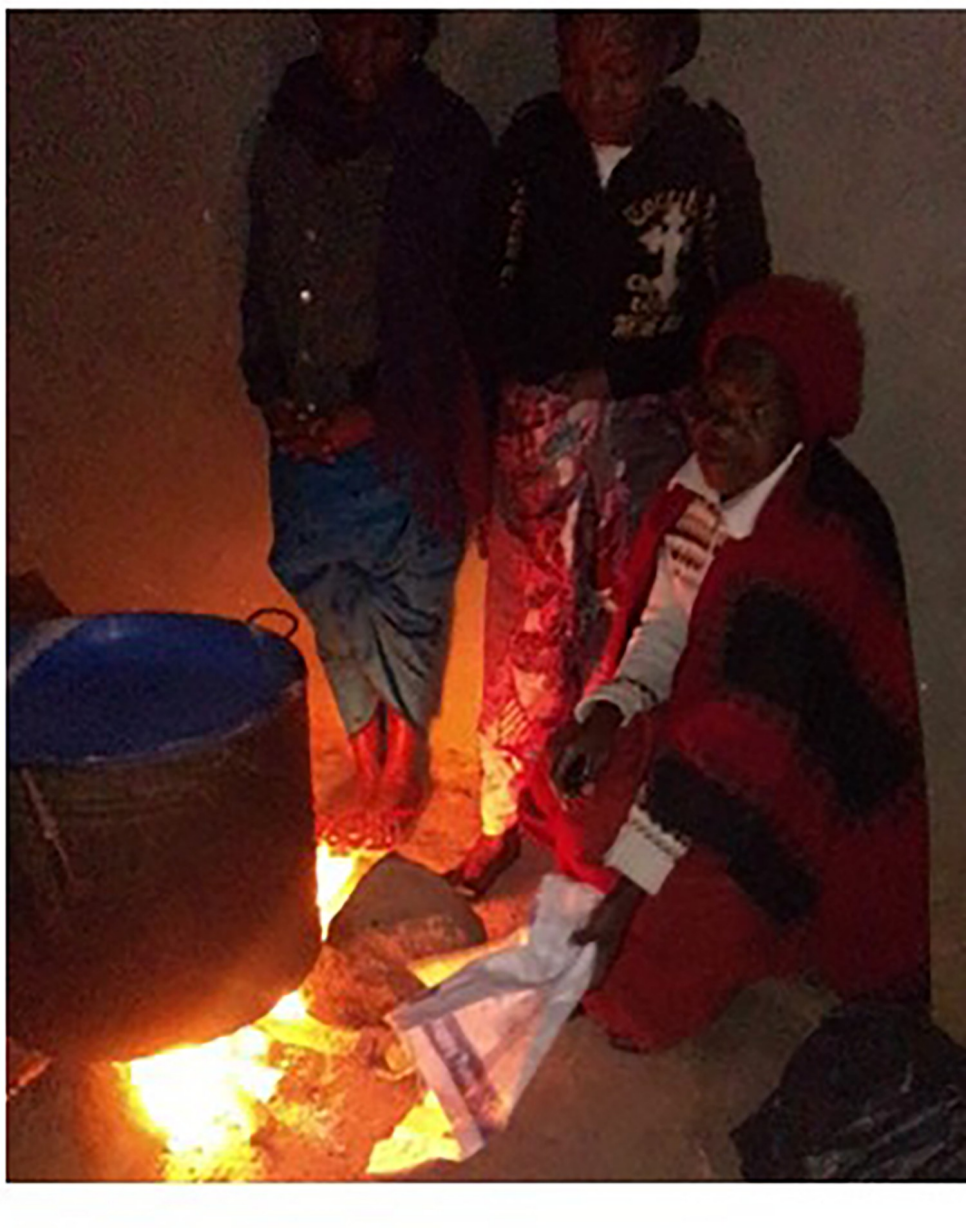
Cooking inside is sometimes needed.

However, some participants also reported that community residents often had no other choice but to use those fuels indoors due to seasonal, social or other environmental factors. As one explained whilst describing his picture presented in Fig 10:

“*There was woman heating water using firewood inside the house, surrounded by her children who had to prepare for school. When i asked her about the large cloud of the smoke in the house, she told me she had no other choice.”(M05; female)*

#### Simple behaviours used to reduce exposure to smoke and HAP

**Fig 11:**
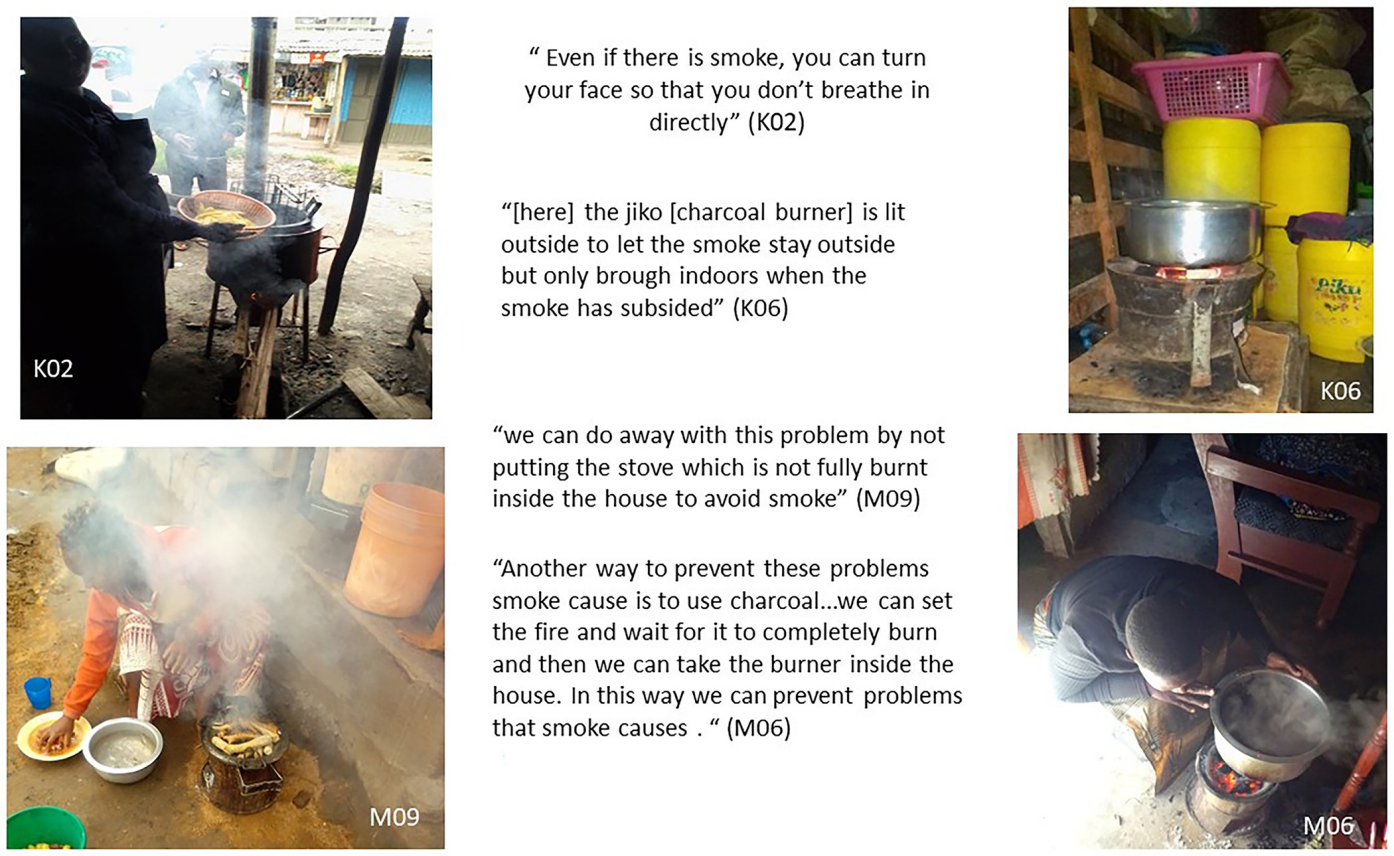
Tactics to reduce exposure to smoke.

Our findings showed that community residents in both slums were not only aware of the many dangers posed by smoke emanating from cooking on solid fuels, but that they also tried in their own ways to reduce the risk to them and their children, where this was feasible. For instance, some explained that they simply tried to move away from the path of the smoke when lighting fires for cooking, or to shield theirs or their children’s face with a cloth (as shown in Figs 7, 8 and 11). Some also attempted to cook outdoors where possible and then only brought the fuel burner indoors when the smoke had subsided. However, lighting fires outdoors for cooking was described as difficult in Mukuru due to the layout and crowdedness of houses and the fact that houses often had no cooking spaces or dedicated kitchens.

Moreover, participants from both slums in this study expressed that some households make decisions around fuel choices which can minimise the risk from exposure to smoke, where they can.

#### Smoke makes everything dirty

**Fig 12:**
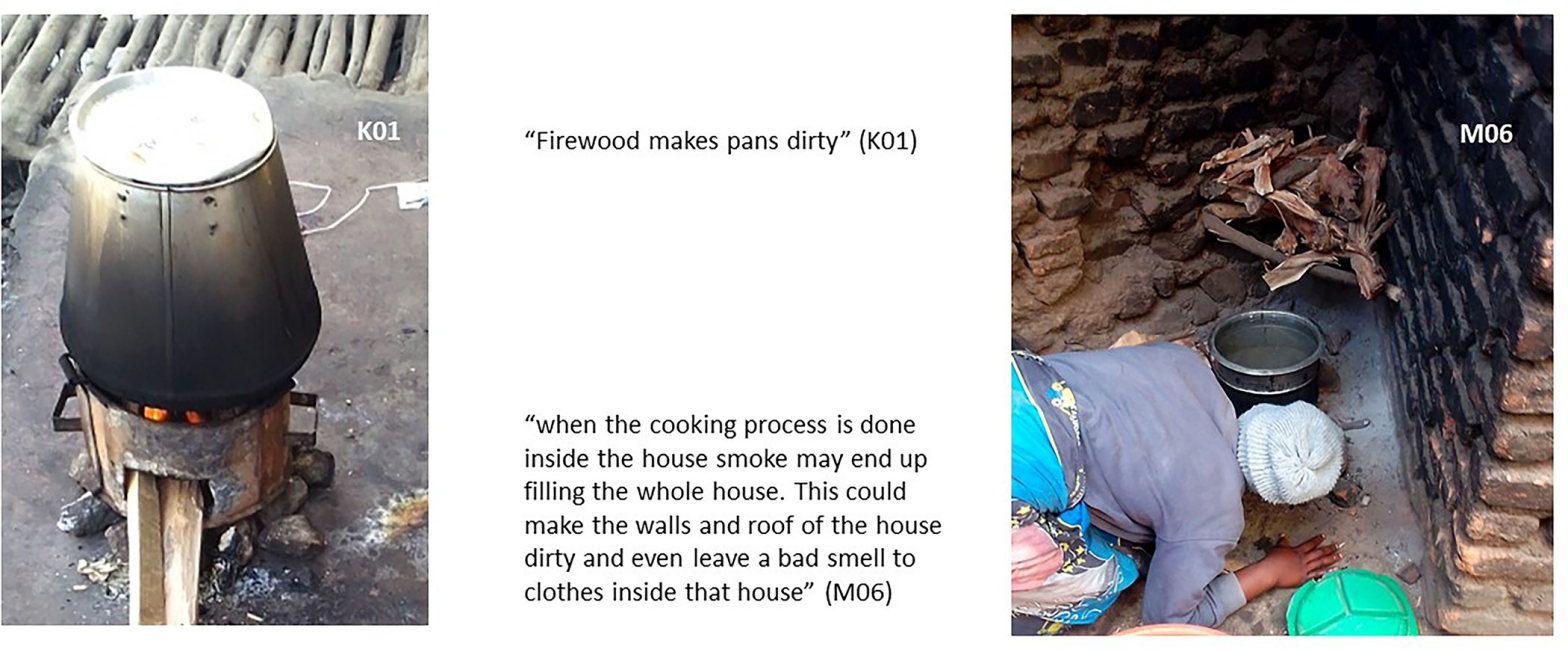
Smoke dirties pots and houses.

Finally, participants mentioned that the smoke and black soot that solid fuels create make houses and cooking pots dirty, which is hard to clean and is seen as unsanitary (as shown in Fig 12). For that reason, participants stated that where they can residents tend to cook outside the house when they can, especially if using fuels that create thick smoke (such as maize waste, firewood, plastic bottles).

### Roles and responsibilities for procuring fuel in the home

**Fig 13:**
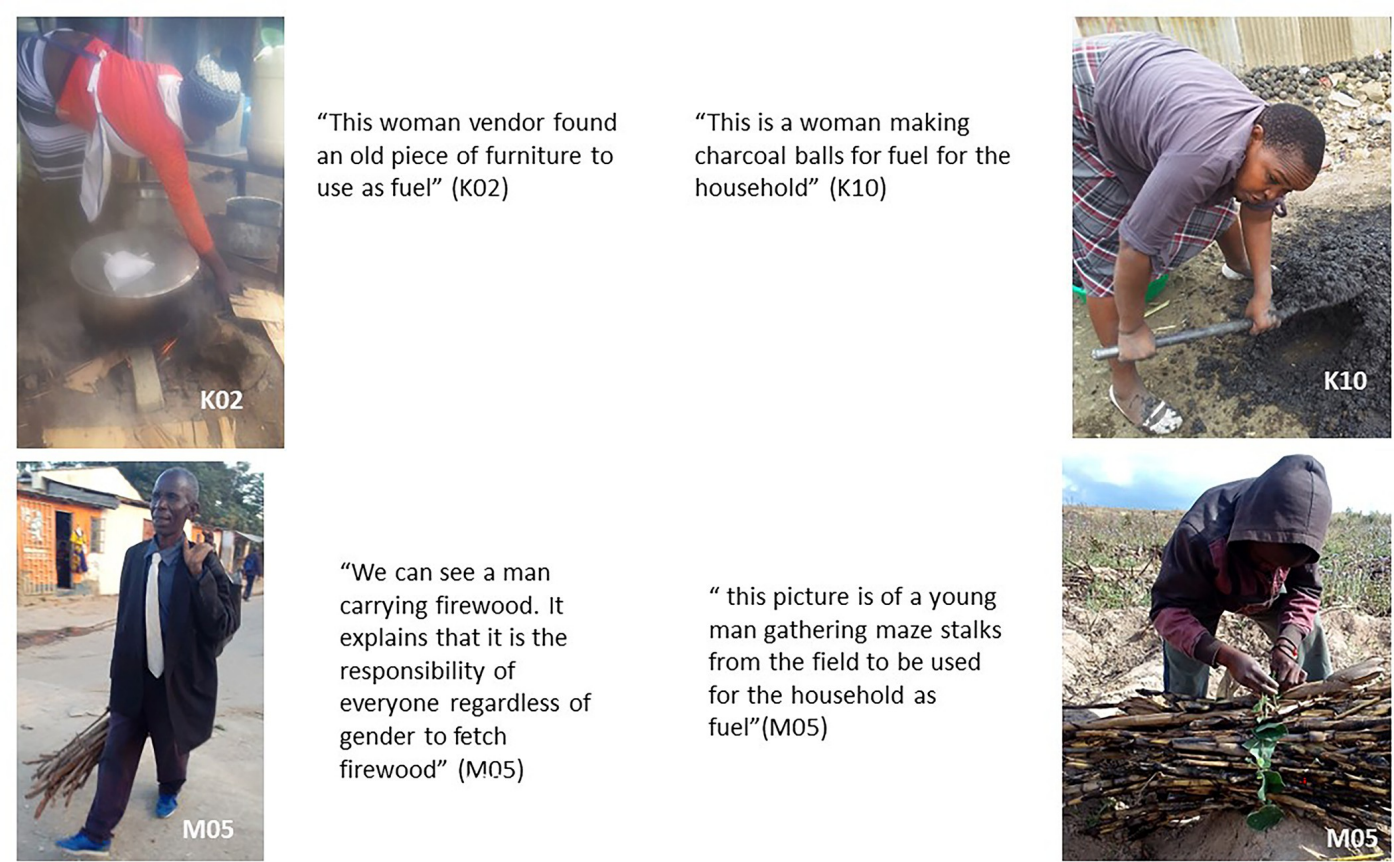
Household roles in fuel procurement.

This photovoice study revealed key insights regarding household members’ responsibility to choose, collect, purchase or use solid fuels in Mukuru and Ndirande. The participants indicated that all family members, regardless of gender, are involved and responsible for making, collecting or purchasing fuel for use at the household level (see Fig 13 below).

Children or young people were often involved in such tasks, especially when parents or guardians were busy working. This was mostly seen as a good thing as it ensures continuity and consistency as a participant expressed:

“*I think it’s good and it should continue because if we say, this task is for men and this one for women a day might come when one would be sick, and this will affect the whole house. People might sleep on empty stomach because someone responsible for buying firewood is sick.”* (M04; female)

However, some participants in Ndirande also mentioned that they were aware of the perceptions by others in the community, who see gathering fuel as the responsibility of women, and underrate men who take part in collecting fuel.

### What are the solutions to reduce the use of solid fuels and who is responsible?

At stages 4 and 5 of our photovoice study process, participants were asked to reflect and discuss whether things could be improved in terms of solid fuel use for cooking in their communities, and improving health, and they suggested their own solutions. They also discussed who they believe has the power to facilitate any changes.

In Mukuru, participants stated that cleaner fuels are already more common with increasing numbers of residents fully or partially using LPG gas and electricity for their cooking needs. The participants there explained that local NGOs had started projects in the slum to reduce use of charcoal and had distributed gas burners and gas cylinders to some residents. They explained that LPG gas could be metered and refilled using mobile phone credit as needs arise (‘pay-as-you-go’). Participants in Mukuru expressed that more education about the health impacts of some solid fuels should be carried out at community level. They stated that this could be done by community members themselves (residents’ groups, landlords), community leaders, local NGOs and other stakeholders. However, they also stated that the responsibility for improving houses and spaces for cooking (e.g. ventilation, dedicated kitchens), as well for making cleaner fuels more affordable, lied with the Government -both at City Council (Nairobi) and at National level-and landlords, as a lot of Mukuru residents are tenants renting their houses.

In Ndirande, where the use of cleaner fuels is still less common, participants stressed that local chiefs and traditional Authorities, as well as the district and national Government, have the power to provide solutions to the problems created by solid fuel use- for instance by regulating the use of charcoal- to mitigate health harms to the population. Some expressed that the Government needs to provide access to clean fuels such as affordable and reliable electricity supplies to all residents. As a participant summarised:

“*The government has the capacity of changing the course of things. If the government can provide cheap or affordable electricity to the people, these problems could be delt with since we will be able to cook without problems. We currently use firewood and charcoal because electricity is expensive. So, if we are provided with affordable/cheap electricity that is available everywhere then it means these problems will be over.” (M08; male)*

## Discussion

This photovoice study is unique in describing in detail- through the eyes of residents themselves- some of the economic, social and cultural determinants of fuel use in the informal settlements of Ndirande and Mukuru. It shows that in those informal settlements firewood and charcoal remain commonly used and highlights the more recent use of charcoal briquettes which are gaining in popularity due to their affordability and ease of use. This study shows also, that due to financial and economic constraints, other ‘fuels of the last resort’-such as maize residue or plastic waste- are used as fuel in those settlements, when households cannot afford to purchase other fuels to cook and heat. Moreover, our findings show that residents are aware of some of the dangers of the smoke arising from the use of these solid fuels to their health, and that they use a range of simple behaviours to try to limit their own exposure and that of their household members. Our exploration of the gender roles in the procurement and use of solid fuels for cooking challenges commonly accepted notions in this area, adding to recent studies in different contexts [50, 54, 64]. Yet such issues require further research, since in informal settlements, women suffer from added vulnerabilities [25]. The added risks to their health posed by cooking on solid fuels disproportionately applies to women who are the primary cooks in some households [12, 26]. For instance, there are impacts of HAP exposure during pregnancy and on birth outcomes which interventions have aimed to address [25]. To our knowledge ours is the first comparative study of solid fuel use and the impact of HAP which used photovoice in both Ndirande and Mukuru.

Our photovoice study demonstrates that informal settlement residents in Mukuru and Ndirande practice fuel stacking and switching, defined as the use of multiple fuels, or a combination of fuels at once- to cover households’ needs for cooking and heating [58–62]. The reality of fuel stacking in such environments challenges the more dated idea that population tend to move along a progressive linear energy ladder from particular solid fuels to cleaner fuels as their household income increases [58, 65]. A recent review of fuel stacking and switching in South Africa [66] shows that instead of moving linearly upward a set energy ladder, the energy choices of the poorest populations are often influenced by their built and social environments as well as their income and other cultural practices. As has been stated in other studies, even as household incomes increase, people may not abandon solid fuels use altogether but rather continue to combine different fuels and cooking stoves and to switch between them [23, 61, 66] according to needs. Relevant evidence shows that fuel stacking and switching may happen for a number of reasons such as: safety (not all environments are safe to gather or use certain fuels in), fuel availability and supply (different fuels are available at different seasons or times), and that the type of food that requires cooking also varies throughout the week and the time available for cooking as well as the type of stoves available to use [58, 62, 67, 68]. Our study echoes all those findings and add more nuanced contextual cultural evidence showing for instance how some foods require certain fuels: for instance where vendors in Mukuru need to heat up snacks fast to sell those to passersby on their way to work at breakfast time and use wood because they can cook several meals for passersby quicker; or in Ndirande, where charcoal is used and preferred to cook maize porridge, which requires prolonged sustained heat. The relationship between fuels used and food preferences, cooking requirements and cultural norms in informal settlements are still poorly studied and require further research. Yet understanding such determinants may be key to developing appropriate and sustainable solutions for health improvements in informal settlements.

Our study further highlights the need to focus HAP and solid fuel research on informal settlement contexts in Africa. Due to high levels of rapid urbanization in SSA, currently approximately 51% of urban populations reside in informal settlements [16]. Those residents are exposed to multiple risks already and they often rely partially or totally on solid fuels for cooking and heating because cleaner fuels (e.g. LPG Gas, electricity) are still either unavailable or unaffordable to them [18–20]. This has a major adverse impact on their health [8, 17, 20, 21]. It is estimated that in 2025 over 1 billion people in sub-Saharan Africa (SSA) will still be relying on polluting fuels [13]. Our study, conducted in two SSA informal settlements clearly reports, in the residents’ own words and pictures, concerns over the smoke and HAP produced from cooking on solid fuels and their associated harms. They also raise concerns about the specific dangers for women and children, who, in informal settlements are more likely to be involved in household related tasks, including cooking and childcare [69, 70]. This added vulnerability of cooking on solid fuels and the harms of HAP exposure disproportionately affect women and young children [9, 71]. For instance, the impacts of HAP exposure during pregnancy and on birth outcomes are well documented [71–73], and a recent quantitative study in urban Nairobi showed that exposure fine particulate matter (PM2.5) peaked for women in the evening, in relation to cooking activities [74]. Another recent study from the large multi-country CLEAN-Air Africa study (taking place in Kenya, Cameroon, Rwanda, Uganda and Tanzania) was the first in SSA to highlight the links between energy poverty and women’s health related quality of life (HRQoL) as well as mental health, for women in peri-urban settings in Cameroon, Ghana and Kenya. They found that longer cooking times, lack of access to electricity and clean fuels as well as being burned or injured while cooking and whilst gathering fuelwood, were significantly associated with women’s lower mental and physical health [74].

Governments in both countries are attempting to address these issues. In Kenya,, the Government has embarked on a strategy to make Liquefied petroleum gas (LPG) the primary clean source of cooking fuel for all so as to phase out solid fuels for environmental and health reasons [75]. The use of LPG has been linked to health improvements, particularly for women in Kenya [76]. However, the target of 35% of the population using LPG by 2030 will remain hard to reach as LPG prices continue to be high. Furthermore, in Kenya, LPG supply is poorly regulated, and the long-term uptake of LPG is not always evaluated. Nonetheless, recently implemented pay-as-you-go (PAYG) payment options for LPG have increased the uptake of LPG in informal settlements [63, 68]. In Malawi, the Government’s new National Energy Policy and the National Charcoal Strategy aim to diversify energy sources, protect the environment from deforestation and increase access to clean energy for all populations [77, 78]. Although electricity is the favoured policy option in Malawi, access and supply remain key barriers [47, 79, 80]. A very recent Government publication expressed that even though 14% of Malawian households now have a national grid connection, less than 2% of the population are primarily cooking with electricity [47], possibly due to unreliable supply (powercuts are common). Moreover 82% of the population still use charcoal as part of their cooking fuel stack, despite the Government attempts to make this illegal to protect the environment and population health [47, 78].

Our study clearly shows that people in those two informal settlements, in Kenya and Malawi, continue to rely on solid fuels because access to affordable, reliable, sustainable, acceptable clean fuels remain a challenge for residents. In slums contexts- which are common across the Africa continent and likely to remain so, it may be unrealistic to expect a linear or rapid transition from solid fuels to clean fuel sources. Instead, interventions to mitigate the exposure to HAP from solid fuels used for cooking should-be embedded in the actual understanding of these populations’ fuel stacking and switching behaviours and should build on people’s own awareness of the dangers of solid fuel use, as highlighted in our study. We contend that this type of exploration is likely to be better achieved by more participatory research methods such as photovoice and walking interviews as well as through other folk media methods which have worked well before in HAP research and in other areas of health research [21, 30, 41, 81–83]. This will also ensure that interventions focus on what is achievable within the socioeconomic and environmental context of communities [84]. Interventions co-designed with informal residents’ communities are likely to be more feasible and sustainable and may lead to further reductions in HAP exposure and associated health improvements if the spaces most amenable to change can be identified [26, 81, 85].

The 2024 State of Global Air report states that “*The solution to addressing household air pollution is simple and its impact is clear*: *improving access to clean cooking improves health*” [1]. Most interventions to date in SSA, as elsewhere, have focused on increasing populations uptake of cleaner fuels and of cleaner cooking technologies (e.g. Improved Cook Stoves (ICS). Those interventions have been somewhat successful in reducing personal exposure to both PM2.5 (fine inhalable particles in the air that are 2.5 micrometers or less in diameter) and Carbon Monoxide (CO) in populations [85]. The evidence shows significant health gains from HAP interventions focused on improving ventilation in the home or kitchen, for instance through the provision of flues or chimneys for stoves [31, 85, 86]. However, our findings show that house spaces and layouts are likely to be an issue in informal settlement, where some have no dedicated kitchen space and houses are packed), and where a large proportion of residents are tenants who do not own their house and may not be in a position of deciding to structurally alter their homes.

Reviews of the effectiveness of HAP interventions globally, highlight issues of low compliance during and after interventions, as well as a poor understanding of the motivations behind stove and fuel ‘stacking’ or switching in different contexts [31, 87]. Several reviews–including one focused on SSA-stressed that relatively few studies contained components of education and awareness raising or behavioural change [87, 88], despite empirical studies highlighting that this may potentially lead to health improvements and to saving lives (for instance awareness of the dangers of CO [49, 89]. Our study certainly demonstrates an awareness of the issues and dangers amongst informal settlements populations which should be harnessed to develop future interventions.

**Fig 14:**
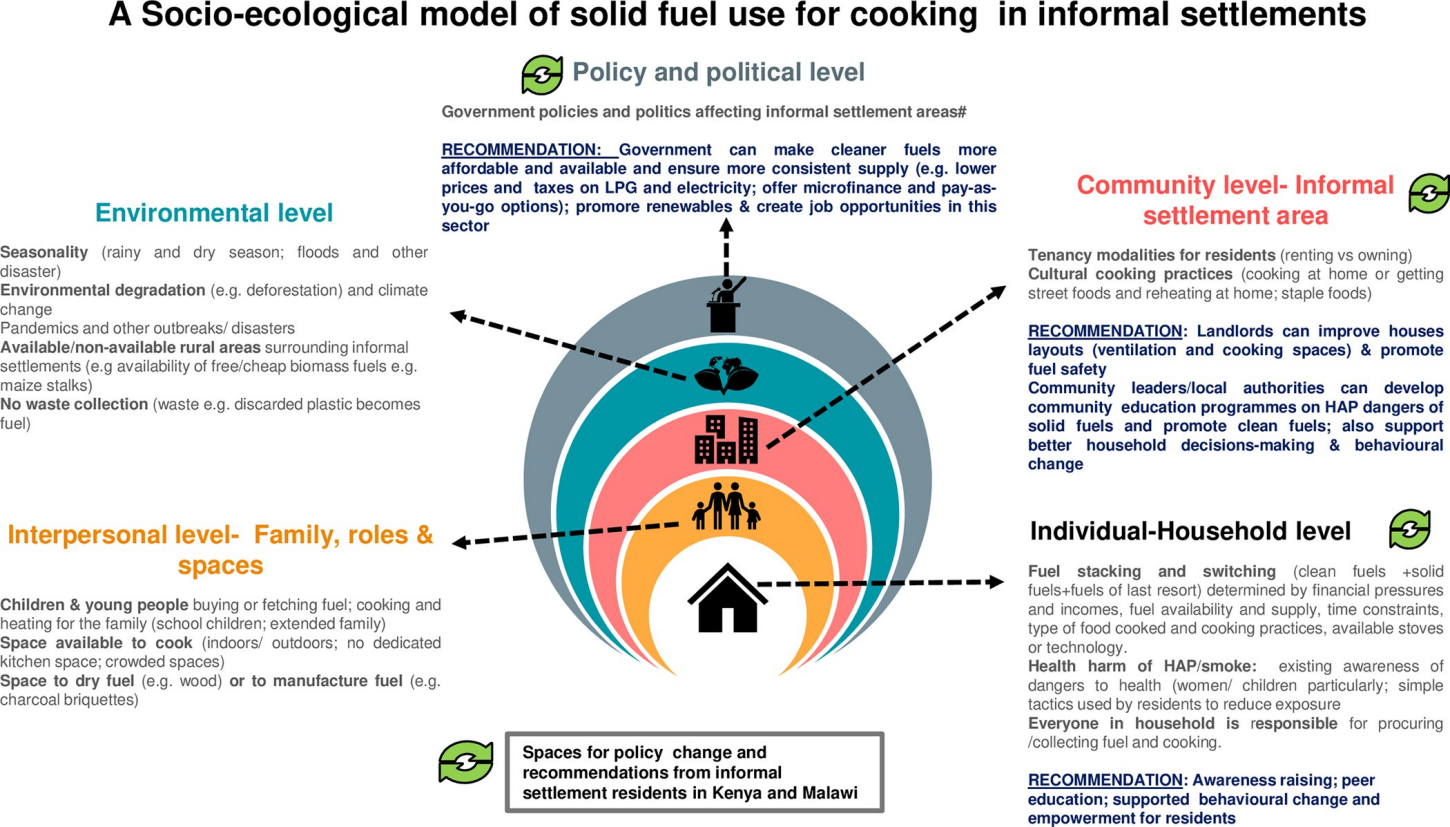
Socio-ecological model of solid fuel use for cooking in informal settlement.

To guide such approaches to future interventions, we build on our previous scoping review [26] and on our own results, to apply Bronfenbrenner’s Social Ecological Model (SEM) [90] in order to offer a new framework for addressing HAP and associated harms from solid fuel use in informal settlements. The SEM, as a framework, suggests that people’s behaviours operate within a number of systems or levels of complex interactions within their households, community and social networks, within their location or environments, and within policy and political systems [90]. In the model below (Fig 14) we situate our findings firmly within this SEM framework and identify the political, environmental, interpersonal, community and individual factors which determine the use of solid fuels in informal settlements. In this diagram, we also highlight spaces (in green) which we believe may be the most amenable to change in the short to medium term, and where we believe interventions could be targeted in these informal settlement and others in Kenya and Malawi. Furthermore, we identify those stakeholders (be them Government officials at local, national and district level; landlords; community leaders or traditional authorities) who are perceived to hold the most power to enact change at all levels In Kenya and Malawi, according to residents in Ndirande and Mukuru. Lastly, we draw on the study to offer simple practical recommendations for action within this model. Even though the contexts of informal settlements can vary from country to country across SSA [17, 70], the issues raised in this paper prevail across a large number of those settlements and we believe that some features may be comparable- particularly the added vulnerabilities suffered by women and children- [17, 70, 91]. Innovations to address HAP and related harms need to apply recently developed gender analysis frameworks which specifically account for gender disparities and gender roles related to energy and health in informal settlements [92]. As such, we believe our methodology, results and model may be applicable and useful to other researchers in Africa, who are concerned with the multifactorial and intersectional dynamics of fuel choices, HAP and population health in informal settlements.

Ensuring the availability and affordability of clean fuels and clean cooking technologies for all falls within the responsibility of governments and multi-sectoral partners, and within the domain of policy change [31, 85, 87]. This transition may take time, meanwhile interventions focused on informal settlements populations- and co-designed with those populations- could mitigate harms, reduce risks and improve health for the most vulnerable populations across sub–Saharan Africa.

### Short reflexivity statement

This short statement is based on the recent guidance by Morton et al [93] around measures promoting equitable partnerships and authorship of papers. This study was developed in collaboration by UK, Kenyan and Malawian researchers who designed the study, jointly applied for funding and have shared the intellectual ownership of the project from the start. All research team members were funded on the study for a percentage of their time in equal measure from 2020 to 2022. The two research assistants (TC, MN) in Malawi and Kenya were employed full time for the duration of the project by the Malawi University for Business and Applied Sciences, and the Kenya Medical Research Institute; they also benefited from a bespoke research capacity training programme throughout the project (covering Nvivo training, qualitative research methods analysis and academic writing). This was aimed at increasing their employability beyond the end of the study period. Research assistants were fully involved in the analysis of the data, and TC was involved in the writing of the paper, thus she features highly in the authorship list. All authors contributed collaboratively to the writing of this paper, the first and last authors are both females at different career stages. We intend to share the published paper with all study participants, who are fully acknowledged in this paper.

### Limitations

The photovoice was conducted in the dry season. This may therefore have contributed to only witnessing particular types of fuels used as available at the time of the study and therefore to the ones pictured in the photovoice. To better understand how solid fuel use choices and behaviours change with seasonality, it would have been useful to repeat the photovoice in the rainy season. However, neither the funding nor the scope of this small exploratory study permitted this. Moreover, the sample for the photovoice is small- as is usually the case for photovoice- [30, 34, 35], primarily for pragmatic reasons because with each participant producing 30 photos each -therefore approximately 300 photos in total to be analysed and sorted by the group at Steps 3 and 4 of our process-, it would be difficult to proceed to a meaningful analysis with a larger sample. Our study was also a 2-year exploratory study with limited time and funding. Involving young people in the photovoice in the informal settlement was difficult- particularly in Mukuru-as residents’ families often rely on them for various activities (e.g. childcare, work), and the study was carried out when schools were in session. As the photovoice training and meetings took place over 3 days at different time points, attendance was possibly easier for young people who are out of school than those in school during the day. To address this, participants were compensated for their travel and time spent away from their chores or work at each and every step of the photovoice.

## Conclusion

This study highlights not only the need to understand the daily life, priorities and concerns of those who use solid fuels on informal settlements, but also the necessity to place them and their experience at the heart of the solutions that will reduce the health harms of HAP. In informal settlements contexts any potential transition to clean fuels is likely to take time and we recommend that in the short-term interventions focus on reducing exposure, supporting harm mitigation and behavioural changes at the household level, whilst continuing to promote cleaner options (e.g. through microfinance [94] or other pay as you go schemes [63, 68]. Advocacy for policy change at Government level will also be required in order to save lives and improve population health for those most at risk in informal settlements.

## Supporting information

S1 File Early thematic framework matrix. (XLSX)
